# Olanzapine With or Without Fosaprepitant for Preventing Chemotherapy Induced Nausea and Vomiting in Patients Receiving Highly Emetogenic Chemotherapy: A Phase III Randomized, Double-Blind, Placebo-Controlled Trial (ALLIANCE A221602)

**DOI:** 10.1093/oncolo/oyad140

**Published:** 2023-06-07

**Authors:** Rudolph M Navari, Jennifer Le-Rademacher, Fabrice Smieliauskas, Kathryn J Ruddy, Thomas James Saphner, Heshan Liu, Elizabeth Harlos, Adedayo A Onitilo, Karthik Giridhar, Preet Paul Singh, Pavan S Reddy, Selina Chow, Flavio Kruter, George Raptis, Charles L Loprinzi

**Affiliations:** Department of Hematology Oncology, Simon Williamson Clinic, Birmingham, AL, USA; Alliance Statistics and Data Management Center, Mayo Clinic, Rochester, MN, USA; Department of Hematology Oncology, Wayne State University, Detroit, MI, USA; Department of Medical Oncology, Mayo Clinic, Rochester, MN, USA; Department of Hematology Oncology, Aurora Cancer Care, Green Bay, WI, USA; Alliance Statistics and Data Management Center, Mayo Clinic, Rochester, MN, USA; Alliance Statistics and Data Management Center, Mayo Clinic, Rochester, MN, USA; Department of Hematology Oncology, Marshfield Clinic-Weston Center, Marshfield, WI, USA; Department of Medical Oncology, Mayo Clinic, Rochester, MN, USA; Department of Hematology Oncology, Springfield Clinic, Heartland NCORP, Springfield, IL, USA; Department of Hematology Oncology, Cancer Center of Kansas, Wichita, KS, USA; Alliance Protocol Operations Office, University of Chicago, Chicago, IL, USA; Department of Hematology Oncology, Carroll Regional Cancer Center, Westminster, MD, USA; Department of Hematology Oncology, Northwell Health Cancer Institute, Lake Success, NY, USA; Department of Medical Oncology, Mayo Clinic, Rochester, MN, USA

## Abstract

**Purpose:**

A protocol was developed to evaluate the value of an NK-1 receptor antagonist for preventing nausea and vomiting resulting from highly emetogenic chemotherapy when an olanzapine-based antiemetogenic regimen was used.

**Materials and Methods:**

A221602, a prospective double-blind, placebo-controlled clinical trial, was developed to compare 2 ­olanzapine-containing antiemetic regimens, one with an NK-1 receptor antagonist (aprepitant or fosaprepitant) and one without. Trial patients had a malignant disease for which they received intravenous highly emetogenic chemotherapy (single day cisplatin ≥ 70 mg/m^2^ or doxorubicin plus cyclophosphamide on 1 day). Patients on both arms received commonly administered doses of a 5-HT_3_ receptor antagonist, dexamethasone, and olanzapine. Additionally, patients were randomized to receive an NK-1 receptor antagonist (fosaprepitant 150 mg IV or aprepitant 130 mg IV) or a corresponding placebo. The primary objective was to compare the proportion of patients with no nausea for 5 days following chemotherapy between the 2 study arms. This trial was designed to test for the noninferiority of deleting the NK-1 receptor antagonist, with noninferiority defined as a decrease in freedom from nausea by less than 10%.

**Results:**

A total of 690 patients were entered on this trial, 50% on each arm. The proportion of patients without nausea for the complete 5-day study period was 7.4% lower (upper limit of the one-sided 95% confidence interval was 13.5%) in the arm without an NK-1 receptor antagonist compared with the arm with an NK-1 receptor antagonist.

**Conclusion:**

This trial did not provide sufficient evidence to support that deletion of the NK-1 receptor antagonist was as good as keeping it, as a part of a 4-drug antiemetic regimen for highly emetogenic chemotherapy (ClinicalTrials.gov Identifier: NCT03578081).

## Teaser

Nausea and vomiting are adverse effects of chemotherapy. This article reports a protocol developed to evaluate the value of an NK-1 receptor antagonist for preventing nausea and vomiting resulting from highly emetogenic chemotherapy with an olanzapine-based antiemetogenic regimen.

**Implications for Practice**

This was a phase III randomized clinical trial for control of chemotherapy-induced nausea and vomiting (CINV) in patients receiving highly emetogenic chemotherapy using olanzapine with or without an NK-1. A previous study demonstrated that adding olanzapine to an NK-1, 5 HT3, and dexamethasone improved control of CINV. The current study considered whether an NK-1 is needed to control CINV if olanzapine is used. This is an important question when deciding the antiemetic regimens prior to chemotherapy.

## Introduction

Chemotherapy-induced nausea and vomiting (CINV), a major adverse effect of cancer treatment, is associated with a significant deterioration in life quality.^1^

In the 1990s, it was demonstrated that the use of ­5-hydroxytryptamine_3_ (5-HT_3_) receptor antagonists plus dexamethasone significantly improved the control of CINV.^1,2^ Subsequent studies demonstrated that additional reduction of CINV occurred with the use of palonosetron, a second generation 5-HT_3_ receptor antagonist^3^ and with the use of neurokinin-1 (NK-1) receptor antagonists.^4,5^ In the 2000s, international guidelines recommended the use of an NK-1 receptor antagonist, a 5-HT3 receptor antagonist, and dexamethasone for prevention of chemotherapy-induced nausea/vomiting related to highly emetogenic chemotherapy regimens.^6-8^

Over the past decade, olanzapine, an antipsychotic medication that blocks multiple neurotransmitters in the central nervous system, has been demonstrated to be an effective agent for preventing CINV.^9-16^ Most notably, in 2016, an Alliance for Clinical Trials in Oncology trial (A221301), designed to evaluate olanzapine for highly emetogenic CINV, demonstrated that olanzapine, when added to a 5-HT3 receptor antagonist, an NK-1 receptor antagonist and dexamethasone, substantially improved the complete response (CR) rate (no vomiting and no use of rescue medications) and markedly improved the number of patients who had no nausea through the 5-day period of time following chemotherapy, when compared with a placebo.^14^ Following this, multiple international antiemetic guidelines recommended olanzapine as an additional agent to prevent ­chemotherapy-induced nausea/vomiting.^6-8^

The above-noted data demonstrate that there are 4 drug types that are effective for decreasing highly emetogenic ­chemotherapy-induced nausea/vomiting (corticosteroids, 5-HT_3_ receptor antagonists, NK-1 receptor antagonists, and the antipsychotic medication olanzapine). This raises a question: can antiemetic therapy regimens for highly emetogenic chemotherapy be de-intensified? Such an approach might decrease side effects from antiemetic agents and decrease financial toxicity, as some of these agents cost hundreds of dollars for each dose.

Given the above question, the current clinical trial was developed to compare 2 olanzapine-containing antiemetic regimens, one with the use of an NK-1 receptor antagonist and one without. When this trial was developed, olanzapine was an inexpensive generic agent while NK-1 receptor antagonists were quite expensive.

## Methods

Alliance A221602 was a prospective double-blind, ­placebo-controlled clinical trial. Eligible patients for this trial were required to have a malignant disease for which highly emetogenic chemotherapy was prescribed. Patients entered on this trial could not have received any previous chemotherapy, must have been at least 18 years old, and must have had an Eastern Cooperative Oncology Group (ECOG) performance status of 2 or better. They could not have had nausea or vomiting in the 24 hours prior to trial registration, a known diagnosis of dementia, a positive pregnancy test, or significant nonmalignant central nervous system disease such as a seizure disorder. In addition, they could not have been receiving treatment with an antipsychotic agent such as olanzapine, risperidone, quetiapine, clozapine, butyrophenone, or a phenothiazine, in the 30 days prior to registration. Also, they could not have received amifostine or quinolone antibiotic therapy within 7 days prior to registration. No radiotherapy was allowed within 7 days prior to registration or planned for 1 week after the initial planned dose of chemotherapy. Patients could not have had chronic alcoholism, a known hypersensitivity to olanzapine, an uncontrolled cardiac arrhythmia, uncontrolled congestive heart failure, an acute myocardial infarction within the previous 6 months, or a history of uncontrolled diabetes mellitus. Adequate renal and liver function was required.

Patients participating in the current trial received intravenous highly emetogenic chemotherapy defined as either (1) cisplatin, given on a single day, at a dose of ≥70 mg/m^2^, with or without other low emetogenic chemotherapy agent(s) on a single day or (2) doxorubicin (60 mg/m^2^) plus cyclophosphamide (600 mg/m^2^) on a single day.

Patients were randomized at a 1:1 ratio to receive an NK-1 receptor antagonist (intravenous [IV] fosaprepitant 150 mg or aprepitant 130 mg), on day one or a corresponding placebo, in a double-blinded manner. Patients were stratified according to gender, their chemotherapy regimen, and the specific 5-HT_3_ receptor antagonist used (ie, palonosetron or ondansetron).

Patients in both arms were scheduled to receive (1) a 5-HT_3_ receptor antagonist (palonosetron 0.25 mg IV or ondansetron 8-16 mg IV or 16-24 mg per oral (PO), allowing clinician choice) on day 1; (2) dexamethasone (12 mg PO, day 1 followed by 8 mg PO, days 2-4), and (3) olanzapine (10 mg/day PO, days 1 to 4, with doses given at approximately 24-hour intervals. On day 1, all agents were to be given prior to chemotherapy, with the exception that olanzapine could have been taken prior to chemotherapy or at bedtime. If the patient did not take the olanzapine prior to chemotherapy and developed nausea or vomiting prior to bedtime, the patient could take it prior to bedtime.

Patients completed a questionnaire at baseline and then daily for 5 days after chemotherapy that inquired about (1) nausea (on a 0-10 numerical analog scale that asked about their worse nausea from “no nausea at all” to “nausea as bad as it can be”; (2) any undesired sedation trouble that they had over the past 24 hours; and (3) any undesired appetite increase that they had over the prior 24 hours.

Patients completed questionnaires daily for 5 days after chemotherapy that also asked them to note, over the prior 24 hours, how much nausea they had, how much vomiting that they had (none, once, twice, or more than twice) and the “number of extra nausea/vomiting pills taken because you developed nausea/vomiting” (none, one, 2, or more than 2).

Nurse phone calls were also made daily for 5 days after chemotherapy to inquire about the same questions indicated above, to encourage the patients to complete their questionnaires, and to address any patient inquiries.

When patients returned for a second cycle of the same chemotherapy, they were encouraged to continue treatment with their initially assigned antiemetic agents for an additional 3 chemotherapy cycles. Those choosing to continue on the protocol were unblinded, given they had completed and submitted all required questionnaires for their first cycle of treatment, and no placebo medication was given. Patients were asked to complete the same questionnaires on the same schedule that was used on the first cycle.

### Statistical Considerations

The study was designed to test whether olanzapine without fosaprepitant/aprepitant was non-inferior to olanzapine with fosaprepitant/aprepitant for prevention of CINV. The primary endpoint was nausea control as defined by the proportion of patients reporting no nausea (a response of 0 in the nausea item of the Nausea and Vomiting Daily Diary/Questionnaire) in the overall (0-120 hours) period. Nausea control in the acute (0-24 hours) and the delayed (24-120 hours) periods were also reported but were only to be tested if olanzapine without fosaprepitant/aprepitant was shown to be noninferior to olanzapine with fosaprepitant/aprepitant (gatekeeping approach). Our previous study^14^ showed that the rate of nausea control in the overall period after treatment with olanzapine + fosaprepitant/aprepitant was 37%. Olanzapine + placebo was to be considered to not be inferior to olanzapine + fosaprepitant/aprepitant if the complete nausea control rate with olanzapine + placebo was less than 10% lower than that with olanzapine + fosaprepitant/aprepitant, ie, a noninferiority margin of 10% was considered clinically meaningful. Assuming the nausea control rate in the olanzapine + fosaprepitant/aprepitant was 37% and using a one-sided Type I error rate of 0.05 with one planned interim analysis for efficacy and futility (non-binding) after 50% of patients had enrolled and completed the Nausea and Vomiting Daily Diary/Questionnaire, 620 patients (310 per arm) were determined to provide 80% power to conclude that olanzapine + placebo was noninferior to olanzapine + fosaprepitant/aprepitant. The total planned sample size was inflated to 690 (345 per arm) to allow for a 10% dropout due to cancellation or major violations. A modified intent-to-treat principle was applied for statistical analysis of efficacy in evaluable patients. Evaluable patients were defined as all patients meeting the eligibility criteria who signed a consent form, started treatment, and had no major violations.

Patient characteristics were summarized by treatment arm. Baseline symptoms were compared between arms using the Kruskal-Wallis test. The primary endpoint of nausea control and secondary endpoints including complete response, undesired appetite increase, and undesired sedation rates were summarized by treatment arm using frequency and proportion. The differences in these rates between treatment arms were estimated (along with 95% confidence interval) and tested using normal approximation of the binomial distribution. Sensitivity analyses of the primary and secondary endpoints using repeated measurement and growth curve models to account for time trend were conducted. Except for the primary endpoint (with a one-sided test of non-inferiority), all confidence intervals and p-values for other endpoints reported were 2 sided. Data collection and statistical analyses were conducted by the Alliance Statistics and Data Management Center. All analyses were conducted using the SAS software version 9.4 on a database locked on January 7, 2022. Data quality was ensured by review of data by the Alliance Statistics and Data Management Center and by the study chairperson following Alliance policies.

This phase III trial was monitored by the Alliance Data and Safety Monitoring Board, a standing committee composed of individuals from within and outside of the Alliance.

## Results

### Randomization and Baseline Data

**Figure 1.**
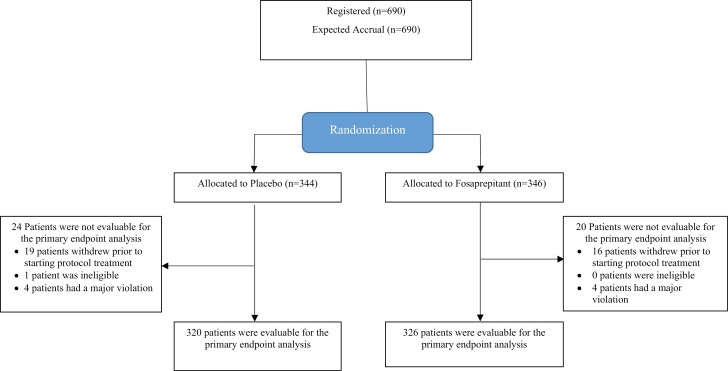
CONSORT diagram.

Patients were entered on this clinical trial from 111 clinical sites between October 2018 and July 2021. Each participant signed an IRB-approved, protocol-specific informed consent document in accordance with federal and institutional guidelines. Data regarding the distribution and randomization of the 690 entered patients are illustrated in a CONSORT diagram (Fig. 1). Demographic data and patient characteristics are well balanced between study arms (Table 1). The majority of patients on this trial were women with breast cancer who received doxorubicin and cyclophosphamide as adjuvant therapy. A total of 646 patients (93.6%) reached the primary endpoint and were evaluable for data analysis.

**Table 1.** Baseline characteristics.

		Arm		
		Placebo (*N* = 344)	Fosaprepitant (*N* = 346)	Total (*N* =690)
Age (years)				
Mean		56.1	56.5	56.3
Median		58.0	58.0	58.0
Range		27.0, 79.0	29.0, 81.0	27.0, 81.0
Race				
White		278 (80.8%)	292 (84.4%)	570 (82.6%)
Not Hispanic		259 (75.3%)	277 (80.1%)	536 (77.7%)
Hispanic		7 (2.0%)	3 (0.9%)	10 (1.4%)
Hispanic origin unknown		12 (3.5%)	12 (3.5%)	12 (3.5%)
Black or African American		40 (11.6%)	36 (10.4%)	76 (11.0%)
Asian		12 (3.5%)	4 (1.2%)	16 (2.3%)
American Indian or Alaskan Native		2 (0.6%)	0 (0.0%)	2 (0.3%)
Native Hawaiian or Pacific Islander		0 (0.0%)	1 (0.3%)	1 (0.1%)
More Than One Race		1 (0.3%)	1 (0.3%)	2 (0.3%)
Unknown or Not reported: patient refused or not available		11 (3.2%)	12 (3.4%)	23 (3.3%)
ECOG Performance Score				
0		281 (82%)	276 (80%)	557 (81%)
1		59 (17%)	67 (19%)	126 (18%)
2		4 (1%)	3 (1%)	7 (1%)
Chemotherapy regimen				
Doxorubicin and cyclophosphamide		266 (77%)	267 (77%)	533 (77%)
Cisplatin-containing regimen		78 (23%)	79 (23%)	157 (23%)
Sex				
Female		289 (84%)	289 (84%)	578 (84%)
Male		55 (16%)	57 (17%)	112 (16%)
5-HT receptor antagonist				
Ondansetron		90 (26%)	90 (26%)	180 (26%)
Palonosetron		254 (74%)	256 (74%)	510 (74%)

Baseline nausea and vomiting questionnaire				
	Arm			
	Placebo (*N* = 344)	Fosaprepitant (*N* = 346)	Total (*N* = 690)	*P*-value
Worst nausea over the past 24 hours (continuous)				.9800^1^
*N*	327	334	661	
Mean (SD)	0.2 (0.74)	0.3 (1.17)	0.2 (0.98)	
Median	0.0	0.0	0.0	
Range	0.0, 5.0	0.0, 10.0	0.0, 10.0	
Undesired appetite increase over the past 24 hours (continuous)				.5687^1^
*N*	327	334	661	
Mean (SD)	0.2 (1.06)	0.2 (0.93)	0.2 (1.00)	
Median	0.0	0.0	0.0	
Range	0.0, 10.0	0.0, 10.0	0.0, 10.0	
Undesired sedation trouble over the past 24 hours (continuous)				.5039^1^
*N*	327	334	661	
Mean (SD)	0.2 (0.81)	0.3 (1.15)	0.2 (1.00)	
Median	0.0	0.0	0.0	
Range	0.0, 6.0	0.0, 10.0	0.0, 10.0	

^1^Kruskal-Wallis *P*-value.

There were no differences between the 2 study arms with regard to the patients’ symptoms over the 24 hours prior to receipt of their chemotherapy as it related to their (1) worst nausea scores, (2) an undesired increase in their appetite, or (3) undesired sedation.

### Efficacy Data

**Figure 2.**
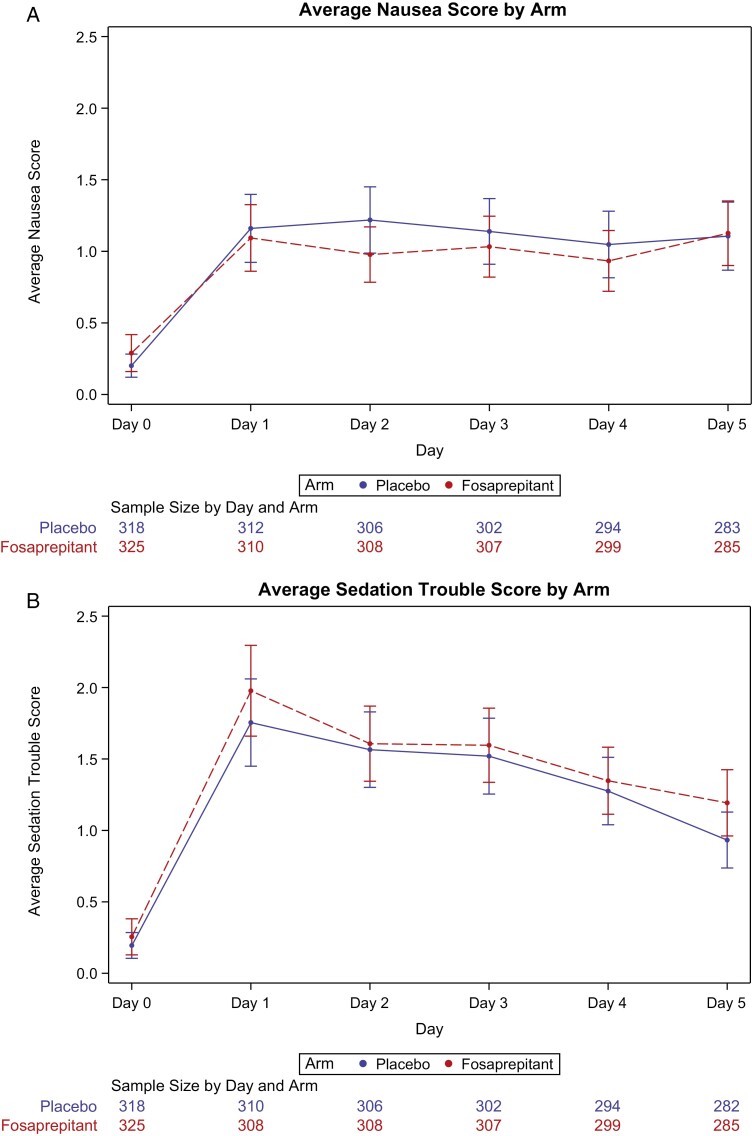
Daily nausea (**A**) and sedation (**B**) Scores by arm in the first cycle.

Table 2 provides data regarding the primary endpoint of this trial, that being that the patient did not have any nausea for 5 days following receipt of their chemotherapy. These data are illustrated in Fig. 2. While there was no suggestion of outcome differences on day 1 (acute period, first 24 hours following chemotherapy) between the 2 study arms, a lower proportion of patients (7.4%, with the upper limit of the one-sided 95% confidence interval being 13.5%) in the arm without fosaprepitant/aprepitant reported no nausea for the complete 5-day study period. Per the study design, set up to exclude a 10% benefit for fosaprepitant, the results did not provide sufficient data to reject the null hypothesis that the 3-drug regimen was inferior to the 4-drug regimen (*P* = .24, for one-sided test of non-inferiority with 10% margin). If the study had been designed to illustrate that fosaprepitant/aprepitant added significant benefit to the other 3 used antiemetic agents, in terms of no nausea for 5 days, the obtained data would have concluded that it did so, with a one-sided superiority test *P*-value of .02.

**Table 2.** Freedom from nausea for the first chemotherapy cycle.

	Placebo (*N* = 320)	Fosaprepitant (*N* = 326)	Estimated difference: Fosaprepitant - placebo (95% One-sided CI)	*P*-value^*^
Overall period (primary endpoint)			0.074 (−inf, 0.135)	.2443
No nausea	97 (30%)	123 (38%)		
Any nausea	223 (70%)	203 (62%)		
Acute period			−0.008 (−inf, 0.054)	Per the gatekeeper procedure specified in the protocol, hypothesis tests for the acute period and the delayed period were not conducted since the null hypothesis for the overall period has not been rejected
No mausea	201 (63%)	202 (62%)		
Any nausea	119 (37%)	124 (38%)		
Delayed period			0.073 (−inf, 0.136)	
No nausea	114 (36%)	140 (43%)		
Any nausea	206 (64%)	186 (57%)		

^*^Non-inferiority test with observed test statistics $z=(\hat{p_{f}}\;\hat{p_{p}}-\;\;\;0.10/\sqrt{\hat{p}(1-\hat{p})[1/n_{f}+1/n_{p}]})=0.661$ where the subscript f denotes fosaprepitant arm and p denotes placebo arm.

One of the secondary endpoints of this trial was to evaluate complete responses (ie, no vomiting and no use of rescue antiemetic treatments) between the 2 study arms. Complete response rates increased from 47% in the placebo arm to 55%% in the fosaprepitant arm (*P* = .0497). Supplementary Tables S1 and S2 provide more details.

Upon completion of the first cycle of therapy, patients were allowed to continue to be treated with the antiemetic regimen that they received with their first cycle for up to 4 total cycles of chemotherapy. Seventy-one percent of patients in each arm agreed to continue with the antiemetic regimen that they initially received. The nausea scores for the subsequent 3 cycles, for the 2 study arms, showed similar patterns to what is observed in Fig. 2.

There was no suggestion that patients that received palonosetron, as opposed to ondansetron, had any improvement in outcomes regarding nausea (*P* = .65) or in the number of rescue antiemetic agents taken (*P* = .86). The specific 5-HT_3_ receptor antagonist used, a stratification factor in the randomization, was chosen by the individual clinicians, as opposed to being dictated by the clinical protocol.

Supplementary Table S3 provides data regarding no nausea, complete response, and no vomiting according to chemotherapy type and sex. We intentionally did not include any *P* values since these data are mainly descriptive and it is a questionable practice to include *P* values for unplanned subgroup comparisons.

### Toxicity

Given data that support that olanzapine can increase both appetite and sedation, these 2 items were examined in the current trial. There were no evident differences between the 2 treatment regimens with regards to undesired appetite increases. When sedation differences between the 2 study arms were examined, there did appear to be more sedation in the first 24 hours in the arm that included the NK-1 receptor antagonist (Fig. 2B; *P* = .017). No other substantial toxicity was reported.

## Discussion

The current study failed to demonstrate non-inferiority, in terms of nausea control, when the NK-1 receptor antagonist was not used in combination with olanzapine, a 5-HT_3_ receptor antagonist, and dexamethasone in patients receiving highly emetogenic chemotherapy. Rather, including the NK-1 receptor antagonist appears to improve the incidence of 5-day freedom from nausea by approximately 7%.

A secondary endpoint in the current trial regarding complete responses (ie, no vomiting and no use of additional antiemetics) also supports that the NK-1 receptor antagonist component of the 4-drug regimen provides a small benefit in the delayed period. This finding is consistent with the demonstrated benefit of NK-1 receptor antagonists for the prevention of emesis in the delayed period postchemotherapy. There was no suggestion of any difference in complete responses between the 2 regimens during the one-day acute period postchemotherapy.

The data demonstrate that fosaprepitant may cause some increased sedation, supporting that 2 of the 4 drugs in this antiemetic regimen (olanzapine and fosaprepitant) cause some sedation.

The Alliance for Clinical Trials in Oncology has now conducted 2 randomized, double-blind, placebo-controlled trials that have studied the 4-drug antiemetic regimen that was used in the current trial. While the current trial took away the NK-1 receptor antagonist from the 4-arm regimen, our previously reported trial took away olanzapine from this 4-arm regimen.^14^ This allows us to compare the benefits and toxicities of olanzapine verus the NK-1 receptor antagonist as parts of the 4-drug antiemetic regimen evaluated in these 2 studies, understanding that caution is appropriate for cross-study comparisons, even when one of the study arms (the 4-drug arm) was identical in both studies and even when the 2 studies were conducted by the same study team.

**Figure 3.**
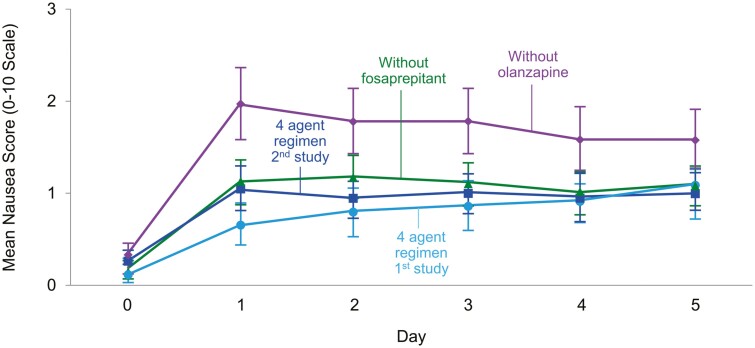
Comparison of daily nausea scores by arm in the 2 arms of the current trial and 2 arms of a previous trial.

Fig. 3 illustrates this comparison, demonstrating that the lowest (best) 2 arms represent data from the 4-drug regimens in each study. This figure also suggests that the results of the 3-drug arm, which includes olanzapine, appears to be better than the 3-drug regimen that includes fosaprepitant/aprepitant. Supplementary Table 1 provides outcomes regarding this 4-arm cross-study comparison for complete responses (no emetic episodes and no use of rescue medication) during the acute, delayed and the overall periods. These data also support that olanzapine better protects against chemotherapy-induced nausea/vomiting than does fosaprepitant.

## Conclusion

The results of this trial support that the studied 4-drug antiemetic regimen is best at preventing nausea and vomiting associated with highly emetogenic chemotherapy. Cross-study comparison suggests that olanzapine may be a more effective antiemetic agent than any available NK-1 receptor antagonist. This is important information for sites that cannot obtain NK-1 receptor antagonists at a reasonable economic price.

## Supplementary Material

oyad140_suppl_Supplementary_Tables

## Data Availability

The data underlying this article will be shared on reasonable request to the corresponding author.
