# Supplementary material for: Olanzapine With or Without Fosaprepitant for Preventing Chemotherapy Induced Nausea and Vomiting in Patients Receiving Highly Emetogenic Chemotherapy: A Phase III Randomized, Double-Blind, Placebo-Controlled Trial (ALLIANCE A221602)
Source: Oncologist. 2023 Jun 7;28(8):722–9. doi: 10.1093/oncolo/oyad140 (PMC10400142; doi:10.1093/oncolo/oyad140)
Supplement: oyad140_suppl_Supplementary_Tables [file oyad140_suppl_supplementary_tables.docx]

# Supplemental Table 1. Complete Response Rates

|  | **Placebo (N=320)** | **Fosaprepitant (N=326)** | **Total (N=646)** | **Estimated Difference (95% CI)** | **P-value**^1^ |
| --- | --- | --- | --- | --- | --- |
|  | **yes (%)** | **yes (%)** | **yes (%)** |  |  |
| **Complete Response –**  **Overall** | 151 (47%) | 179 (55%) | 330 (51%) | 0.077 (0,0.154) | .0497 |
| **Complete Response –**  **Acute** | 247 (77%) | 256 (79%) | 503 (78%) | 0.013 (-0.051,0.077) | .6817 |
| **Complete Response –**  **Delayed** | 166 (52%) | 193 (59%) | 359 (56%) | 0.073 (-0.003,0.150) | .0609 |
| ^1^ Chi-Square *P*-value | | | | | |

**Supplemental Table 2.** Complete Response Comparisons for Both Alliance Studies: Complete response (no emetic episodes and no use of rescue medication) during the acute, delayed, and the overall periods.

|  | **Trial A221301-First Randomized trial (with or without olanzapine) [14]** | | **Trial A221602-Second randomized trial (with or without fosaprepitant*)** | |
| --- | --- | --- | --- | --- |
|  | **3 drug regimen which included fosaprepitant**  **(N=188)** | **4 drug regimen**  **(N= 192)** | **4 drug regimen  (N=326)** | **3 drug regimen which included olanzapine (N=320)** |
| **Complete Response - Overall** | 41% | 64% | 55% | 47% |
| **Complete Response - Acute** | 65% | 86% | 79% | 77% |
| **Complete Response - Delayed** | 52% | 67% | 59% | 52% |

* or aprepitant

**Supplemental Table 3**. Nausea, Response, and Vomiting by Sex and Chemotherapy

**No Nausea-Overall Period**

| **Proportion with no nausea** | **Placebo (n=320)** | **Fosprepitant (n=326)** |
| --- | --- | --- |
|  | | |
| **Chemotherapy Regimen** | | |
| Anthracycline and cyclophosphamide (n=508) | 31% (78/251) | 38% (97/257) |
| Cisplatin-containing (n=138) | 28% (19/69) | 38% (26/69) |
|  | | |
| **Sex** | | |
| Female (n=548) | 30% (81/271) | 38% (105/277) |
| Male (n=98) | 33% (16/49) | 37% (18/49) |

**Complete Response-Overall Period**

| **Proportion with complete response** | **Placebo (n=320)** | **Fosprepitant (n=326)** |
| --- | --- | --- |
|  | | |
| **Chemotherapy Regimen** | | |
| Anthracycline and cyclophosphamide (n=508) | 49% (123/251) | 53% (136/257) |
| Cisplatin-containing (n=138) | 41% (28/69) | 62% (43/69) |
|  | | |
| **Sex** | | |
| Female (n=548) | 46% (126/271) | 53% (147/277) |
| Male (n=98) | 51% (25/49) | 65% (32/49) |

**No Vomiting-Overall Period**

| **Proportion with no vomiting episode** | **Placebo (n=320)** | **Fosprepitant (n=326)** |
| --- | --- | --- |
|  | | |
| **Chemotherapy Regimen** | | |
| Anthracycline and cyclophosphamide (n=508) | 71% (177/251) | 81% (209/257) |
| Cisplatin-containing (n=138) | 58% (40/69) | 70% (48/69) |
|  | | |
| **Sex** | | |
| Female (n=548) | 69% (186/271) | 79% (220/277) |
| Male (n=98) | 63% (31/49) | 76% (37/49) |
